# Limited Value of Single Sampling for IgM Antibody Determination as a Diagnostic Approach for Acute Hepatitis E Virus Infection

**DOI:** 10.1128/spectrum.00382-21

**Published:** 2021-07-07

**Authors:** Antonio Rivero-Juarez, Pedro Lopez-Lopez, Juan Antonio Pineda, Juan Carlos Alados, Ana Fuentes-López, Encarnación Ramirez-Arellano, Carolina Freyre, Ana Belén Perez, Mario Frias, Antonio Rivero

**Affiliations:** a Unit of Infectious Diseases, Hospital Universitario Reina Sofia, Instituto Maimónides de Investigación Biomédica de Córdoba (IMIBIC), Universidad de Córdoba (UCO), Cordoba, Spain; b Unit of Infectious Diseases and Microbiology, Hospital Universitario de Valme, Seville, Spain; c Clinical Microbiology Unit, Hospital Universitario de Jerez, Cádiz, Spain; d Clinical Microbiology Unit, Hospital Universitario Clínico San Cecilio, Granada, Spain; e Instituto de Investigacion Biosanitaria Ibs.Granada, Granada, Spain; f Infectious Diseases, Microbiology and Preventive Medicine Unit, Virgen Macarena University Hospital, University of Seville/Biomedicine Institute of Seville, Seville, Spain; g Department of Medicine, University of Seville/Biomedicine Institute of Seville, Seville, Spain; h Clinical Microbiology Unit, University Hospital of Puerto Real, Cádiz, Spain; i Clinical Microbiology Unit, Hospital Universitario Reina Sofía, Instituto Maimónides de Investigación Biomédica de Córdoba (IMIBIC), Córdoba, Spain; University of Sussex

**Keywords:** hepatitis E virus, ELISA, PCR, diagnosis, IgM, acute hepatitis, diagnostics

## Abstract

The objective was to evaluate the accuracy of a single determination of IgM antibodies for hepatitis E virus (HEV) diagnosis in patients with acute hepatitis. A prospective study included patients with suspicion of HEV infection, defined as individuals with acute hepatitis showing negative results for serological and molecular markers of other hepatitis viruses. All patients were evaluated for hepatitis E virus infection, including both IgM antibodies and viral RNA determinations. Hepatitis E virus infection was defined as positivity for any of these markers. A total of 182 patients were included in the study, of whom 68 (37.4%) were diagnosed with HEV infection. Of these, 29 (42.6%) were positive for both IgM and HEV RNA, 25 (36.8%) were positive only for IgM antibodies, and 14 (20.6%) were positive only for HEV RNA. Considering only those individuals who were positive for IgM antibodies, 54 of the 68 total cases (79.4%) could be identified, showing a percentage of false-negative individuals of 20.6%. The diagnostic algorithm of hepatitis E virus infection in patients with acute hepatitis should include the determination of both IgM antibodies and HEV RNA because single sampling for IgM antibody determination led to an important proportion of misdiagnosed cases.

**IMPORTANCE** In immunocompetent patients with a suspicion of hepatitis E virus (HEV) infection, single IgM antibody testing is typically applied. In this prospective study, we aimed to evaluate the accuracy of three different HEV screening approaches in patients with acute hepatitis, including approaches based on IgM determination, HEV RNA detection, and the combination of both. Our study shows that any diagnostic algorithm for HEV infection in patients with acute hepatitis should be based on the determination of both markers (IgM antibodies and HEV RNA) because single sampling for IgM antibodies results in an unacceptable number of false-negative results (20%). According to our results, the determination of HEV RNA should not be limited to immunosuppressed individuals because a high proportion of cases could be misdiagnosed.

## INTRODUCTION

Hepatitis E virus (HEV) is recognized as a major cause of acute hepatitis in Europe and worldwide (1, 2). During the acute phase, different serological markers can be applied for diagnosis. Viral RNA can be detected between 2 and 6 weeks before the onset of symptoms and is undetectable in serum approximately 3 weeks later (3). Conversely, the immune response follows a transient increase in IgM antibodies, which are detected during the acute phase of the disease and may last up to 12 months (3). The most commonly used approach for the diagnosis of HEV infection is testing for IgM antibodies by an enzyme-linked immunosorbent assay (ELISA). This approach is widely implemented due to its advantage of being easy to implement at a low cost. In contrast, the determination of HEV RNA requires a more complex procedure and, consequently, an increase in the cost of screening. Nevertheless, the main advantage is that it has a higher specificity than the determination of IgM antibodies, with a higher sensitivity during the first days of the onset of symptoms (1, 3). Thus, European HEV guidelines recommend using a combination of serology and HEV RNA testing by PCR to diagnose acute HEV infection (1). However, this recommendation is not supported by an evaluation study; consequently, the determination of IgM antibodies remains the only diagnostic approach in the majority of settings (2). Thus, we aimed to evaluate the accuracy of HEV diagnosis in patients with acute hepatitis, including approaches based on single sampling for IgM determination and HEV RNA detection.

## RESULTS

During the study period, 182 patients with suspected HEV infection were included in the study. Of them, 94 (51.6%) were male, and the median age was 49 years (interquartile range [IQR], 37 to 56 years). A total of 68 (37.4% [95% confidence interval {CI}, 30.3% to 44.4%]) patients were diagnosed with HEV infection. Of them, the majority were male (*n* = 46; 67.6%), and the median age was 47 years (IQR, 37 to 55 years). The baseline characteristics of patients are shown in Table 1. Three patients were infected by HIV, all of whom had undetectable HIV loads and CD4^+^ cell counts of >200 cells/ml.

**TABLE 1:** Baseline characteristics of patients with a diagnosis of Hepatitis E virus infection

Characteristic^*a*^	Value
No. (%) of male patients	46 (67.6)
Median age (yrs) (IQR)	47 (37–55)
No. (%) of patients with hospital admission	8 (11.7)
No. (%) of patients with underlying condition	
HIV infection	3 (4.4)
Diabetes mellitus	2 (2.9)
Chronic hepatitis B	1 (1.4)
Pregnancy	1 (1.4)
No. (%) of patients with symptom	
Fever	34 (50)
Digestive^*b*^	34 (50)
Articular pain	23 (33.8)
Jaundice	21 (30.8)
Limb pruritus	9 (13.2)
Analytical parameter value [median (IQR)]	
ALT (U/liter)	131 (36–435)
AST (U/liter)	97 (23–396)
GGT (U/liter)	115 (35–286)
Total bilirubin (mg/dl)	0.7 (0.6–4.6)

a ALT, alanine aminotransferase; AST, aspartate aminotransferase; GGT, gamma-glutamyltransferase.

b Digestive symptoms include vomiting, diarrhea, and abdominal pain.

Forty-three (63.2%) patients showed detectable HEV loads, and their strains were sequenced. All strains were consistent with genotype 3, and most of them were classified as genotype 3f (90.6%). Only four sequences were consistent with other subtypes, including one classified as genotype 3e (GenBank accession number MN628559), another classified as genotype 3m (GenBank accession number MT250083), and two cases in which a subtype could not be assigned (GenBank accession numbers MT776550 and MT250081).

Of the 68 cases of HEV infections, 54 out 68 cases (79.4%) were positive for IgM. Of them, 29 (42.6%) were positive for both IgM and HEV RNA, and 25 (36.8%) were positive only for IgM. Finally, 14 (20.6%) patients with detectable HEV RNA exhibited negativity for IgM antibodies. The accuracy of hepatitis E virus diagnosis based on the single consideration of IgM antibodies or HEV RNA at the same time point is shown in Table 2.

**TABLE 2:** Accuracy of hepatitis E virus diagnosis based on single consideration of IgM antibodies or HEV RNA^*a*^

Diagnostic approach	No. of positive patients/no. of cases of HEV infection (%)	No. of negative patients/no. of cases of HEV infection (%)	AUROC (95% CI)^*b*^
Anti-HEV IgM	54/68 (79.4)	14/68 (20.6)	0.89 (0.83–0.95)
HEV RNA	43/68 (63.2)	25/68 (36.8)	0.81 (0.74–0.88)

a HEV, hepatitis E virus; AUROC, area under the receiver operating characteristic curve; CI, confidence interval.

b The presence of anti-HEV IgM or HEV RNA (any positive) is considered a reference approach and includes all cases of HEV infection (68/68; AUROC = 1).

Regarding IgG antibodies, 38 out 54 (70.3%) patients with positivity for IgM antibodies also exhibited positivity for IgG antibodies. None of the patients with detectable HEV RNA were positive for IgG antibodies.

## DISCUSSION

The European Centers for Disease Control (ECDC) considers the determination of HEV RNA in acute cases optional because PCR testing might not be available in all laboratories and settings (2). Thus, in immunocompetent patients with a suspicion of HEV infection, single IgM antibody testing is typically applied (4). HEV RNA testing is applied only for diagnosis in immunocompromised subjects given the delay or lack of antibody seroconversion (5). Our study shows that any diagnostic algorithm for HEV infection in patients with acute hepatitis should be based on the determination of both markers (IgM antibodies and HEV RNA) because single sampling for IgM antibody determination results in an unacceptable number of false-negative results. According to our results, the determination of HEV RNA should not be limited to immunosuppressed individuals because a high proportion of cases could be misdiagnosed. In this sense, our study provides evidence that supports the European Association for the Study of the Liver (EASL) recommendation to use both ELISAs and PCR as screening approaches in patients with a suspicion of HEV infection (1).

The sensitivity and accuracy of a screening approach based on single sampling for IgM antibody determination will depend on the assay employed (4). In our study, we used one of the commercial kits with higher sensitivity and specificity, which consequently is one of the most commonly used kits worldwide (6). Using this assay, we failed to detect 20% of the confirmed cases of HEV infection by HEV RNA detection and sequencing in our study. Thus, the sensitivity of this assay could be much lower than previously considered (approximately 80%), which is consistent with previous reports (7, 8). This point strengthens the fact that HEV RNA determination should be included in screening for acute HEV infection.

Our study presents several limitations. First, only patients with acute hepatitis were included. HEV infection can result in extrahepatic manifestations, even in the absence of liver damage (9). Consequently, the determination of both markers in screening for extrahepatic HEV infection needs to be evaluated. Second, only cases of HEV infection by genotype 3 were detected. Thus, the accuracy of this screening approach needs to be evaluated in other settings where other genotypes are circulating. Third, in the present study, we evaluated HEV RNA only in serum and did not consider stool samples. Because the virus is shed in feces for a long period, the use of both serum and stool samples could significantly increase the diagnostic value of PCR determination. Nevertheless, we cannot evaluate this because stool is not included in sampling for screening for acute hepatitis. Fourth, our study is based on single sampling for IgM antibody determination. Because there is a delay between detectable HEV RNA and IgM seroconversion at early phases of the infection, testing serial samples for IgM could increase the diagnostic value of this screening approach. Finally, these results could vary if other ELISAs and PCR protocols are employed.

In conclusion, our study provides evidence that in the diagnostic algorithm of HEV infection in patients with acute hepatitis, the determination of both IgM antibodies and HEV RNA is necessary. The single use of one of these markers could lead to an important proportion of misdiagnosed cases.

## MATERIALS AND METHODS

### Population.

A prospective study was conducted in 6 reference hospitals in Andalusia (South Spain) between February 2016 and November 2020, including patients with suspicion of HEV infection. These patients were diagnosed with acute hepatitis and were negative for other hepatitis viruses, including serological and molecular markers for hepatitis A virus (IgM antibodies), hepatitis B virus (HBsAg, HBcAc, and viral DNA), hepatitis C virus (IgG antibodies and viral RNA), cytomegalovirus (IgM antibodies), and Epstein-Barr virus (IgM antibodies).

### HEV evaluation.

The same serum sample was evaluated for hepatitis E virus infection, including both IgM antibodies and viral RNA. IgM antibodies were evaluated by an enzyme immunoassay using the HEV-IgM kit developed by Wantai (Beijing Wantai Biological Pharmacy Enterprise Ltd., Beijing, China) with an automated procedure (Triturus; Grifols), and positivity was confirmed by immunoblotting (recomLine HEV IgG/IgM; Mikrogen Diagnostik, Neuried, Germany). Additionally, in all patients, IgG antibodies were determined using the specific Wantai kit (Beijing Wantai Biological Pharmacy Enterprise Ltd., Beijing, China), also confirming positive results by immunoblotting. For HEV molecular analysis, RNA was extracted from 400 μl of serum using the QIAamp Mini Elute virus spin kit (Qiagen, Hilden, Germany) by an automated procedure (QIAcube; Qiagen). The purified RNA was eluted in a 50-μl volume. For reverse transcription-quantitative PCR (RT-qPCR), the Qiagen one-step PCR kit (Qiagen, Hilden, Germany) was used for 25 μl of the template (50-μl reaction volume) following a pangenotypic in-house protocol targeting the open reading frame 3 (ORF3) region developed and validated by our group, with the detection limit set at 21 IU/ml (10). Samples positive for HEV RNA were sequenced by nested RT-PCR targeting the ORF2 region according to a procedure described previously (10). Subtype assignment and phylogenetic analyses were performed using the HEVnet genotyping tool (https://www.rivm.nl/mpf/typingtool/hev/) and confirmed by BLAST analysis (11).

### Statistical analysis.

Hepatitis E virus infection was considered positive in an individual exhibiting positivity for IgM antibodies and/or detectable HEV RNA according to the definitions of clinical guidelines (1, 3). The frequency of HEV genotype distributions was reported.

### Ethics statement.

This study was designed and conducted in accordance with the Declaration of Helsinki. The Ethics and Clinical Trials Committee (CEIC) of Andalusia approved the study protocol, obtaining informed consent from each patient (reference number 4535). The SSPA Biobank coordinated the collection, processing, handling, and assignment of the biological samples used in this study according to standard procedures established for this purpose (agreement number S2100110).

### Data availability.

All data generated or analyzed during the study are included in the article. The data sets used and/or analyzed during the present research project are available from the corresponding author upon reasonable request. All sequences were submitted to GenBank (accession numbers MN628557 to MN628567, MT250081, MT250082, MT250083, MN537838, MN914126, MN914127, MT776550 to MT776554, MT854329, and MW143072).
